# A biomechanical mechanism for initiating DNA packaging

**DOI:** 10.1093/nar/gku896

**Published:** 2014-10-01

**Authors:** Haowei Wang, Samuel Yehoshua, Sabrina S. Ali, William Wiley Navarre, Joshua N. Milstein

**Affiliations:** 1 Department of Chemical and Physical Sciences, University of Toronto Mississauga, Mississauga, ON L5L 1C6, Canada; 2 Department of Physics, University of Toronto, Toronto, ON M5S 1A7, Canada; 3 Department of Molecular Genetics, University of Toronto, Toronto, ON M5S 1A8, Canada

## Abstract

The bacterial chromosome is under varying levels of mechanical stress due to a high degree of crowding and dynamic protein–DNA interactions experienced within the nucleoid. DNA tension is difficult to measure in cells and its functional significance remains unclear although *in vitro* experiments have implicated a range of biomechanical phenomena. Using single-molecule tools, we have uncovered a novel protein–DNA interaction that responds to fluctuations in mechanical tension by condensing DNA. We combined tethered particle motion (TPM) and optical tweezers experiments to probe the effects of tension on DNA in the presence of the Hha/H-NS complex. The nucleoid structuring protein H-NS is a key regulator of DNA condensation and gene expression in enterobacteria and its activity *in vivo* is affected by the accessory factor Hha. We find that tension, induced by optical tweezers, causes the rapid compaction of DNA in the presence of the Hha/H-NS complex, but not in the presence of H-NS alone. Our results imply that H-NS requires Hha to condense bacterial DNA and that this condensation could be triggered by the level of mechanical tension experienced along different regions of the chromosome.

## INTRODUCTION

Bacterial chromosomes are highly dynamic entities. On evolutionary timescales, the genomes of bacteria are continuously scrambled through extensive horizontal gene transfer and gene loss (1–3) . On shorter timescales, epigenetic and transient regulatory mechanisms guide cellular function. Take, for example, the various topological conformations displayed by the bacterial chromosome. The bare chromosomal DNA of *Escherichia coli* would have a radius of gyration of ∼5 μm, which is significantly larger than the size of the cell (1–2 μm). It is no wonder then that conformational changes in bacterial DNA, arising from compaction and crowding, are involved in a multitude of cellular processes. The interior of a cell is also a complex and active environment with fluctuations driven by a combination of thermal, entropic and non-equilibrium phenomena. It has been suggested that the entropic forces, which such a tightly confined polymer would exhibit, are an important factor in chromosome organization and segregation and lead to, for example, the spontaneous de-mixing of daughter strands during cell division (4). In fact, we are just beginning to understand how the biomechanical and dynamical properties of the chromosome affect cell function.

It remains unclear how functional order can be maintained in genomes that are constantly remodeling both their content and topology. It is presumed that bacterial nucleoids are not arrayed randomly within the cell, but rather are spatially organized in a manner that enables precise control of critical functions like replication, repair and gene expression, and that this arrangement is robust in the face of rapidly changing and unpredictable external environments (5,6). Toward this end, *E. coli* harbors a number of proteins that appear to be critical to the organization of its nucleoid including HU, IHF, Fis, LeuO, MukBEF and the heat-stable nucleoid binding protein H-NS (7–9).

H-NS acts as a global transcriptional silencer and is known to control the expression of over 400 genes in both *E. coli* and *Salmonella* through a variety of mechanisms (e.g., by affecting DNA topology to constrain supercoiling) (10–14). H-NS preferentially binds AT-rich sequences in the bacterial chromosome, most of which have been acquired via horizontal gene transfer. As a result of its activity, H-NS functions as the master regulator of xenogeneic (foreign-derived) sequences including many of those involved in bacterial virulence (15).

H-NS has three discrete domains: an N-terminal dimerization domain, a central dimerization domain and a C-terminal DNA binding domain (16–18). Three-dimensional crystal structures have revealed that H-NS can form filaments via interactions between the dimerization domains giving rise to extended super-helical structures (16). These findings suggest that the DNA–protein complex may generate or stabilize plectonemes within the bacterial chromosome (8,16). Likewise, single-molecule studies have indicated that H-NS, when bound to DNA, forms stiffened rod like filaments and may also condense DNA through *trans* binding or bridging of adjacent strands of DNA (19–22). The parameter that seems to most influence whether H-NS will stiffen or bridge DNA is the concentration of divalent cations (notably, Mg^2+^) in the assay buffer (22), where low concentrations lead to stiffening while concentrations above 2 mM lead to bridging. As we illustrate, cooperative proteins can generate unique bridging modes of H-NS bound DNA even in the absence of Mg^2+^. While there has been much progress in visualizing the cooperative behavior of H-NS, the exact nature of these extended nucleoprotein complexes is still under debate.

Moreover, at most loci, H-NS is unable to silence transcription without the assistance of members of the Hha/YdgT family of co-regulatory proteins. These small (∼8 kDa) proteins associate directly with H-NS through interactions with H-NS's N-terminal dimerization domain (23–26), but the biophysical consequences of their interaction has not been explored. Here we employ single-molecule methods to demonstrate that H-NS in complex with the cofactor Hha, but neither molecule alone, may be triggered to condense single DNA duplexes via the application of an applied mechanical force. This may provide a previously unobserved mechanism for controlling gene expression and organizing/packaging the bacterial genome through biomechanical control.

## MATERIALS AND METHODS

### Protein purification

Hha and H-NS expression constructs were transformed into the *E. coli* BL21 (DE3) strain, and the transformants were selected on Luria-Bertani (LB) agar plates supplemented with 100 μg/ml ampicillin. The resulting strains were grown in liquid culture until an optical density at 600 nm of 0.6 was reached. Isopropyl 1-thio-β-d-galactopyranoside was added to a final concentration of 1 mm, and the induced expression cultures were grown for an additional 16 h at 15°C with shaking. Cells from 1-l cultures were harvested by centrifugation at 2500 × *g* for 30 min and resuspended in 25 ml of cell lysis buffer (10-mm Tris, pH 8.0, 500-mm NaCl, 10-mm imidazole and 5-mm β-mercaptoethanol). The cell suspensions were lysed by sonication, and the insoluble cellular debris was removed by centrifugation at 13,000 × *g* for 45 min. Qiagen Ni^2+^ resin was equilibrated in cell lysis buffer and incubated with the cell lysates for 15 min at 4°C on a rocking platform. The cell lysate/Ni^2+^ resin mixture was applied to a gravity flow column and washed with 50 ml of wash buffer (10-mm Tris, pH 8.0, 500-mm NaCl, 30-mm imidazole and 5-mm β-mercaptoethanol). The proteins of interest were eluted from the Ni^2+^ resin with 15 ml of elution buffer (10-mm Tris, pH 8.0, 500-mm NaCl, 250-mm imidazole and 5-mm β-mercaptoethanol).

Following Ni^2+^ chromatography, all samples were further purified by gel filtration chromatography over a Superdex 200 16/60 column from GE Healthcare, pre-equilibrated with storage buffer (25-mM N-2-hydroxyethylpiperazine-N-2-ethane sulfonic acid (HEPES), pH 7.5, 150-mm NaCl, 5-mm Dithiothreitol (DTT) and 5% (v/v) glycerol). Protein-containing fractions were pooled and analyzed by sodium dodecyl sulphate-polyacrylamide gel electrophoresis. Thrombin was added to purified Hha protein samples for removal of the N-terminal His_6_ tag.

### DNA amplification

All experiments were conducted on an AT rich (57% AT) DNA fragment that was polymerase chain reaction amplified from the gram negative bacteria *Salmonella* about a 5092 bps (∼1.7 μm) region of the chromosome surrounding the *pagC* gene and promoter using primers GT010 (5′-ACCTGATATTGAGTGGCCTGCTGTCGCACAGCTGATTGTTGATAAG) and GT011 (5′-TCATTCAACACCCGCACTATCGGACAGCTTCGCAGGAGATTTCTA). The resulting product was diluted and re-amplified with 20 nt, end-complementary, primers containing 5′ digoxigenin or biotin modifications to enable attachment of the DNA fragment to a microsphere and glass coverslip, respectively.

### Sample preparation

In all experiments, 2 μl of NIST traceable streptavidin coated beads of 1 μm diameter were diluted by 10 μl of phosphate buffered saline (PBS) and sonicated for 5 min. Two microliters of 5 ng/μl DNA, diluted from the PCR amplified product, was mixed with the beads and tumbled at 4°C for 6 h. Microchambers were created by sandwiching Parafilm between an ethanol rinsed glass slide and microscope coverslip. The chambers were washed with PBS, then injected with 50 μl of 20 μg/ml anti-digoxigenin and incubated at 4°C for at least 20 min. The chamber was then washed twice with 100 μl of HKE buffer (25-mM HEPES, 50-mM KCl, 0.5-mM DTT, 0.1-mM ethylenediaminetetraacetic acid, 0.5-mg/ml α-casein, pH 7.5) and left to rest for 5 min. The DNA/bead solution was mixed with 15 μl of 2x HKE buffer, injected into the chamber and incubated at 4°C overnight. Finally, 400 μl of HKE buffer was used to gently wash away any unbound beads.

### Optical tweezers

A 1064 nm wavelength laser was sent through an acousto-optic modulator followed by an optical isolator before being introduced into a high-intensity single mode polarization maintaining fiber. A beam sampler was used to reflect 10% of the laser output from the fiber onto a photodetector, which worked with a proportional-integral-derivative (PID) controller and the acousto-optic modulator (AOM) to feedback stabilize the root mean square (RMS) laser power to 0.5% . The beam was expanded to overfill the back aperture of an Olympus oil immersion PlanApo objective (60x, 1.4 N.A.) mounted on an Olympus IX-71 microscope, which formed the optical trap. The microchamber could be coarsely translated in xy with a microstage and finely positioned in xyz via piezo-control. The scattered laser light was collected by an Abbe condenser (0.4–1.2 N.A.) and the position of the microsphere relative to the trapping laser could be determined by imaging the back focal plane of the condenser onto a quadrant photodiode. All instrumentation was controlled by a custom LabVIEW program.

### TPM microscopy

Bright field images of tethered particles were collected by a CCD camera at 25 Hz. A custom LabVIEW program was used to track the xy coordinates of each tethered particle, which was determined from the centroid of the microsphere. All RMS values were calculated within a moving window of 4 s. The microspheres where screened for multiple-tethers by requiring the circularity of their RMS motion to deviate by no more than 10% and high-pass filtered to remove sample drift.

### Persistence length measurements

The persistence length is a quantitative measure of intrinsic stiffness that defines the length scale at which a semi-flexible polymer changes from being stiff and rod-like to soft and flexible. To infer the intrinsic stiffness from tethered particle motion (TPM) experiments, we must convert the observed root-mean square excursion *R*_RMS_ of a tethered microsphere, in the plane of the sample specimen, to a persistence length. Fortunately, a simple relation is provided by Segall *et* *al*. (27) under the approximation that the DNA behaves as a Gaussian chain, which is a good approximation for unstressed DNA:

(1) \begin{equation*} R_{{\rm RMS}} = \left( {\frac{L_{\rm C} \xi }{ 3}\left( {2 + \frac{{4N_{\rm R} }}{{\sqrt \pi {\rm erf}(N_{\rm R} )}}} \right)} \right)^{1/2} , \end{equation*}

where *L*_C_ is the DNA contour length, *ξ* is the persistence length and the excursion number $N_{\rm R} \equiv R/\sqrt {L_{\rm C} \xi /3}$, with *R* being the radius of the microsphere.

**Figure 1.**
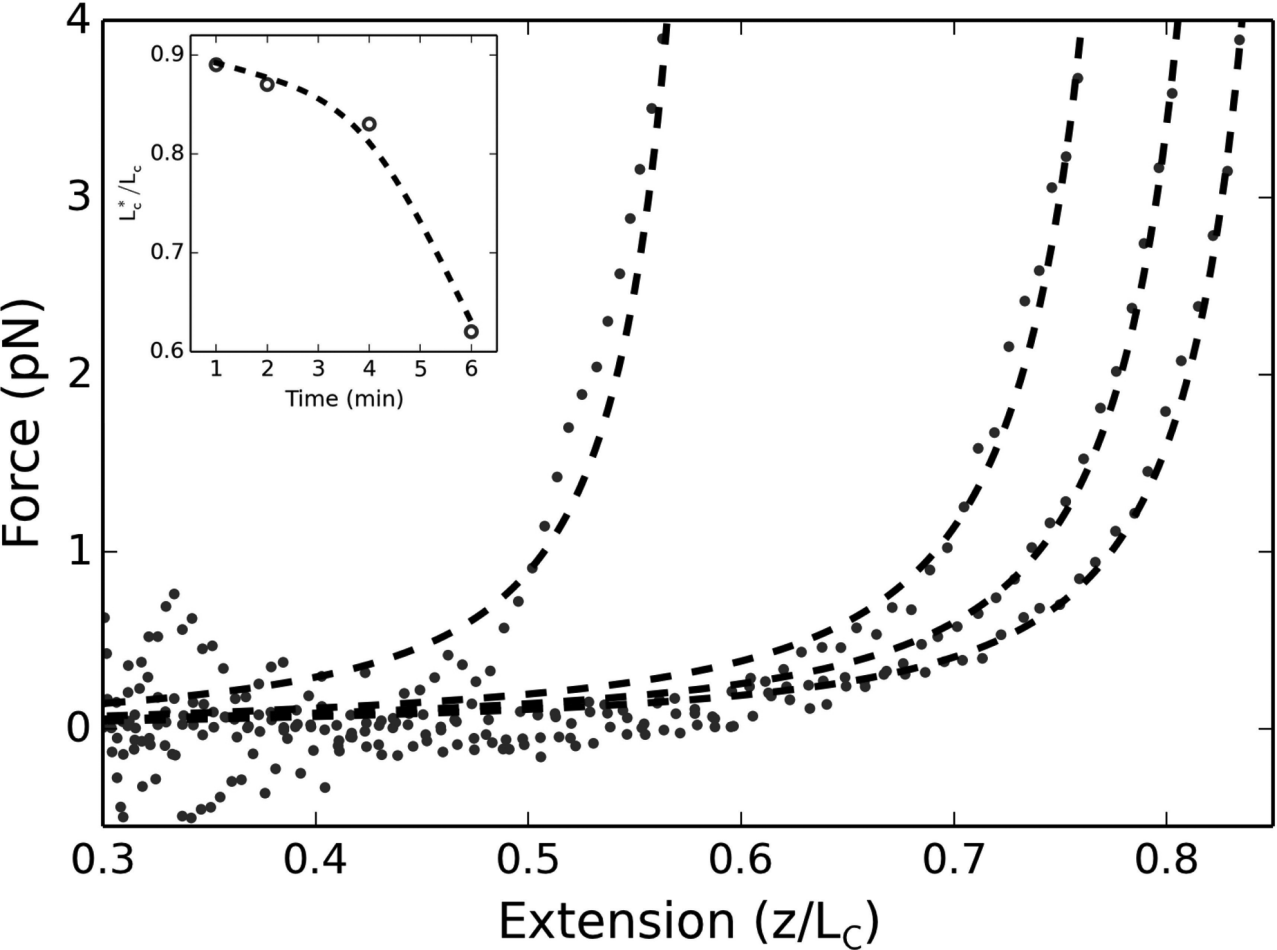
Force-extension curves for 200 nM of H-NS and Hha. From right to left: 1, 2, 4 and 6 min after optically trapping/centering the attached microsphere. Dashed curves are WLC fits with variable DNA persistence and contour lengths. Inset: relative change in contour length obtained by the fits (dashed line is an interpolation).

Alternatively, by applying an optical force, persistence lengths can be extracted from force-extension measurements, as was done in Figure 1, by fitting to the Worm-Like Chain (WLC) model of Marko and Siggia (28):

(2) \begin{equation*} f = \frac{{k_{\rm B} T}}{\xi }\left( {\frac{1}{{4\left( {1 - \frac{z}{{L_{\rm C} }}} \right)^2 }} - \frac{1}{4} + \frac{z}{{L_{\rm C} }}} \right), \end{equation*}

where *f* is the applied force, *z* is the total DNA extension and the Boltzmann factor $k_{\rm B} T = 4.1\;{\rm pN} \cdot {\rm nm}$. The accuracy of extracting a persistence length from TPM measurements can be confirmed by control experiments, without any protein, that compare TPM to force-extension measurements. For our 5-kb DNA, the measured bead motion was $R_{{\rm RMS}} = 508 \pm 13\;{\rm nm}$, corresponding to a persistence length of $\xi = 54 \pm 5\;{\rm nm}$, which is comparable to the persistence length obtained by stretching with optical tweezers $\xi = 53 \pm 4\;{\rm nm}$.

## RESULTS

### Hha/H-NS complex leads to DNA condensation under tension

We performed a series of single-molecule experiments on the Hha/H-NS complex to determine what effect, if any, Hha may have on the ability of H-NS to alter the properties of the DNA to which it is bound. We found Hha alone to have no effect on the DNA. However, we were surprised to find that these two proteins (Hha and H-NS), when bound to the DNA, could be triggered to condense the DNA by the application of a slight mechanical tension. If we failed to apply tension or if the co-regulatory protein Hha was absent, we did not witness condensation.

Optical tweezers were initially used to stretch the DNA in complex with Hha/H-NS to obtain a persistence length, which can serve as a reliable, albeit indirect, indicator of protein–DNA interactions. The persistence length is a quantitative measure of intrinsic stiffness that defines the length scale at which a semi-flexible polymer changes from being stiff and rod-like to soft and flexible. In our experiments, 5 kbp of DNA was surface tethered to a microscope coverslip at one end and affixed to a 1-μm microsphere at the opposite end. The DNA was stretched by optically trapping the microsphere while moving the slide chamber via control of a piezo stage. All experiments were performed with an AT-rich DNA fragment that includes the *Salmonella pagC* gene and promoter, previously shown to bind H-NS *in* *vivo* (10,12). The chamber was flushed with 200 nM of H-NS and an equal concentration of the cofactor Hha, in a Mg^2+^ free HKE buffer, then incubated for 15 min. The tweezers extended the DNA below full extension (above 85% of the contour length) in both directions corresponding to a maximum applied force of ∼10 pN.

Figure 1 displays the force-extension curves at varying times relative to the end of the incubation period. Each curve is measured from high force to low force and then high force again, taking ∼30 s to acquire. Collapse of the protein–DNA complex is indicated by a dramatic shift of the curves to the left over the course of 6 min. Shortly after the initial force-extension measurement, the curves could no longer be fit by a worm-like-chain model if we assumed that a 5 kbp contour length was maintained; however, we were able to generate reliable fits by including the contour length as an additional free parameter (see Supplementary Materials Figure S1 for an error analysis of the fits). Figure 1 is typical of the data we obtained and implies a relatively ordered compaction of the DNA, as the curves consistently shift from right to left. We should note that to obtain force-extension curves requires initially centering the DNA within the optical trap, which is achieved by stretching the DNA a few initial times both along x and y. The procedure takes ∼1 min, which is why our initial force extension curve is obtained 1 min after the incubation period. This technical requirement makes it difficult to control the amount of force we apply to the DNA at *t =* 0 or to use this assay to assess the amount of tension necessary to induce collapse.

We next performed a series of experiments where we briefly triggered DNA condensation and then passively tracked the collapse dynamics by analyzing the motion of the tethered microsphere. This sort of analysis is commonly referred to as TPM microscopy. TPM experiments have provided valuable insight into a variety of biological processes from protein-mediated DNA looping to the procession of RNA and DNA polymerases (29,30). H-NS and Hha were added to the slide chamber in equal concentrations (of either 100 or 200 nM; no effect on DNA was observed at 50 nM) and the system was allowed to reach equilibrium. A single tethered microsphere was selected, then approximately centered within the optical trap before switching on the trap (positioned at ∼1 μm above the coverslip). The laser remained on for 1 min, confining the motion of the microsphere and pulling it toward the laser focus thereby stressing a single protein–DNA complex. The trap was then switched off and the RMS motion of the attached microsphere was tracked for 30 min while concurrently recording the motion of the unaffected tethered DNA within the chamber. This was repeated, sequentially, until all of the selected tethers were induced to collapse. Note, one complication of this experiment is that we do not have precise control over the initial force with which we trigger the collapse. We can only approximately situate the trapped microsphere within the optical potential since the moment we switch on the laser trap, we affect the protein–DNA complex. Nonetheless, with our current approach, we are able to consistently apply forces below ∼3 pN, down to ∼0.5 pN, and find the collapse to be quite robust even at the smallest forces that we apply. Likewise, we stretched the DNA complex for a 1 min duration because this resulted in a robust collapse. Below, roughly, 1 min the probability of collapse was increasingly variable, which is likely coupled to the uncertainty in our applied initial force.

**Figure 2.**
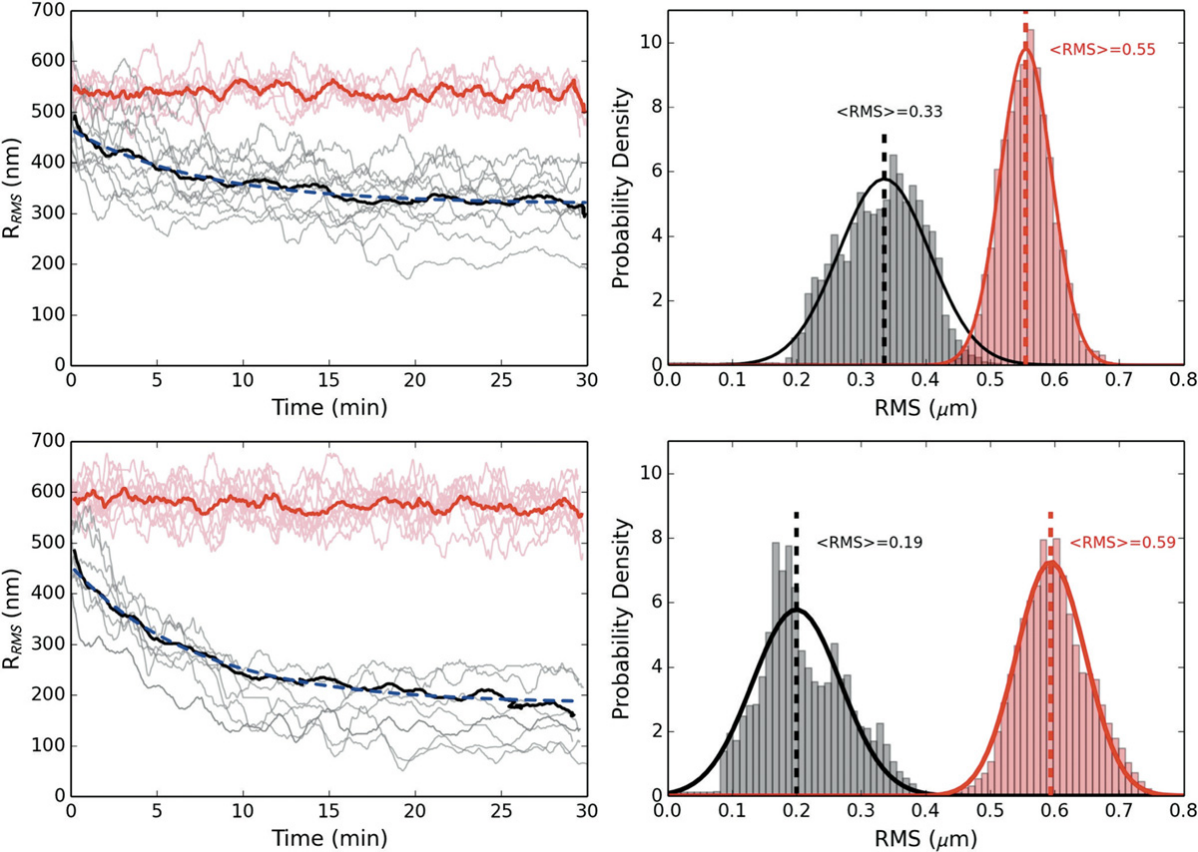
Dynamics of DNA condensation after applied tension. The top (bottom) figure is for equal concentrations of H-NS and Hha of 100 (200) nM. The red curves are the control absent an force applied. The prominent solid lines are averaged over the respective trajectories and fitted by a decaying exponential (dashed/blue) (τ = 7.5 (7.3) min for 100 (200) nM protein). All trajectories have been smoothed by a 60 s running window. Top (bottom) right: RMS histograms for 100 (200) nM protein for control (red) and collapsed (black) population after 15 minutes. The trajectories were fitted to a Gaussian to provide a mean RMS value.

Figure 2 shows the dynamical change in RMS motion of multiple tethers, which are plotted together so that *t* = 0 refers to the moment when each tethered microsphere is released from the optical trap. At a 100 nM concentration of H-NS and Hha, an applied tension triggers a dynamic collapse of the nucleoprotein complex that occurs on the order of 10–15 min. The final RMS are found to be between 1/2 and 1/3 of the pre-stretched RMS. At 200 nM, however, the complex shows much greater compaction, on similar time scales, with some complexes displaying an RMS motion an order-of-magnitude less than before the force was applied. Note that occasionally the tether length shortens because the bead sticks to the surface, but these are rapid, discrete events easily filtered from the data. The relatively flat curves at the top of each figure display the behavior of control tethers that were not stretched by the optical tweezers and show that the protein–DNA complex does not condense in the absence of an applied force. Once collapsed, the protein–DNA complex appears quite stable. We repeatedly tried to extend the collapsed tethers with our optical tweezers, exerting forces of up to 50 pN, but were unable to reverse the collapse.

### Applied force affects H-NS protein DNA interactions

We also considered the effects of tension on DNA bound by H-NS, but absent the co-regulatory protein Hha, with both optical tweezers and, as an alternative way to probe the mechanics (i.e. extract a persistence length) of the H-NS/DNA complex without the addition of an applied force, TPM microscopy. We first measured persistence lengths from the TPM trajectories of individual DNA fragments bound by H-NS prior to the addition of force (pre-stretch TPM). We next stretched these fragments with optical tweezers to generate force-extension curves from which we again extracted persistence lengths, then turned off the trap and determined the persistence lengths a third time by TPM (post-stretch TPM).

**Figure 3.**
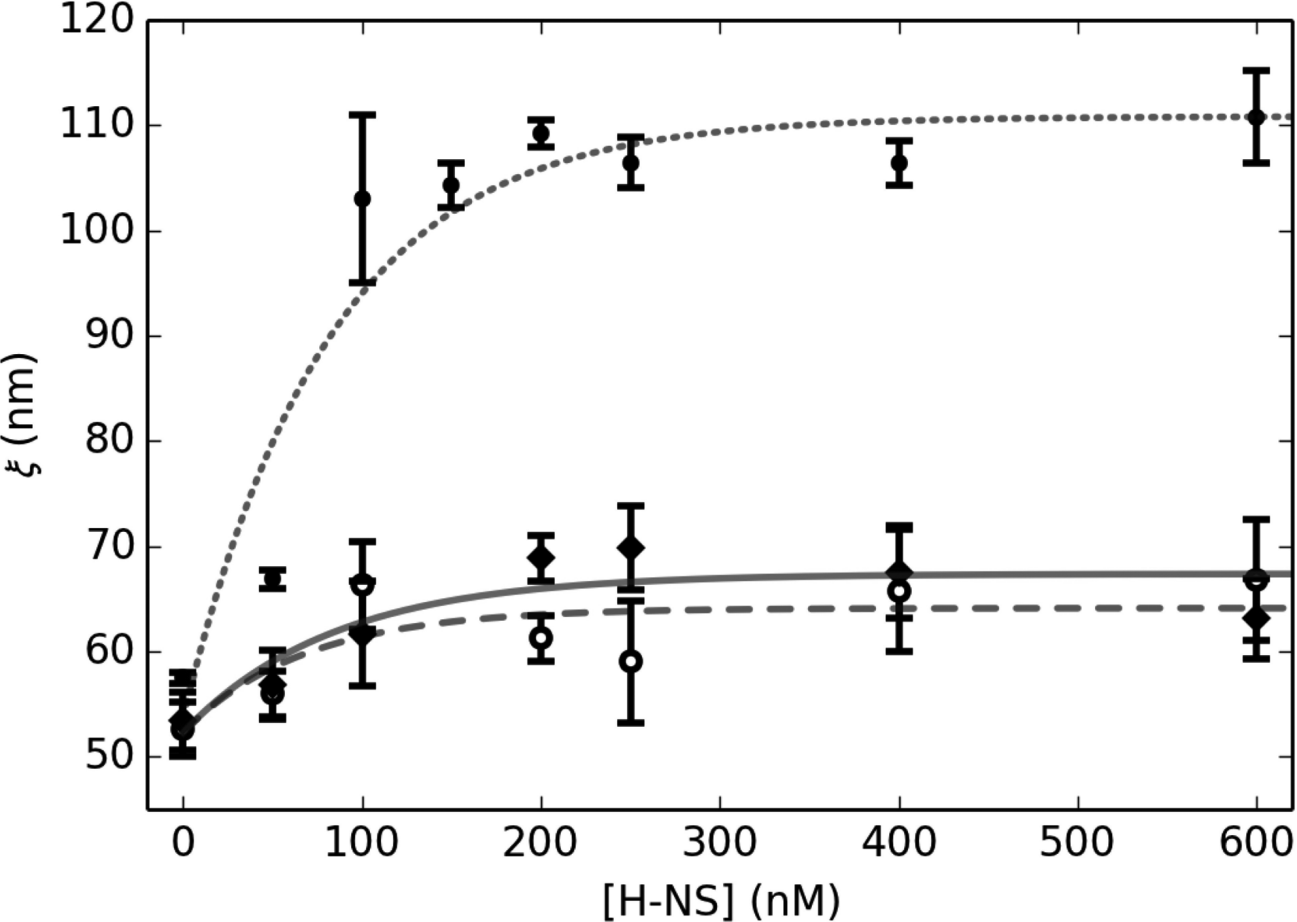
Persistence length dependence upon H-NS concentration. Measured via TPM pre-stressed (circles/dotted line) and post stressed (open circles/dashed line). Measured via force extension with optical tweezers (diamonds/solid line). Exponential fits to the data (lines) provide a reasonable interpolation.

While all the measured persistence lengths increased significantly as a function of H-NS protein concentration, those values obtained by force-extension, although in agreement with previously published force-extension measurements (19), were much lower than those obtained by the pre-stretch TPM measurements. Interestingly, the persistence lengths measured by the post-stretch TPM yielded much lower values, which agreed with the force-extension measurements throughout the range of concentrations (Figure 3). Note, the post-stretch values for the persistence lengths remained stable for at least an hour and did not show any signs of returning to their pre-stretched values. Likewise, by tracking the population of neighboring untouched beads, we could see that the pre-stretch values remained stable throughout the duration of each experiment. We should remark that both our WLC fits and TPM analysis assumed a constant 5-kb contour length. This assumption is supported by the fact that both the post-stretch TPM and the optical tweezers values for persistence length agreed within our measured uncertainty. We also performed a regression analysis of the fits to our force-extension data and found no discernable improvement by including the contour length as an additional parameter (Supplementary Figure S2).

### Mutations in Hha that abolish function *in vivo* prevent DNA condensation

**Figure 4.**
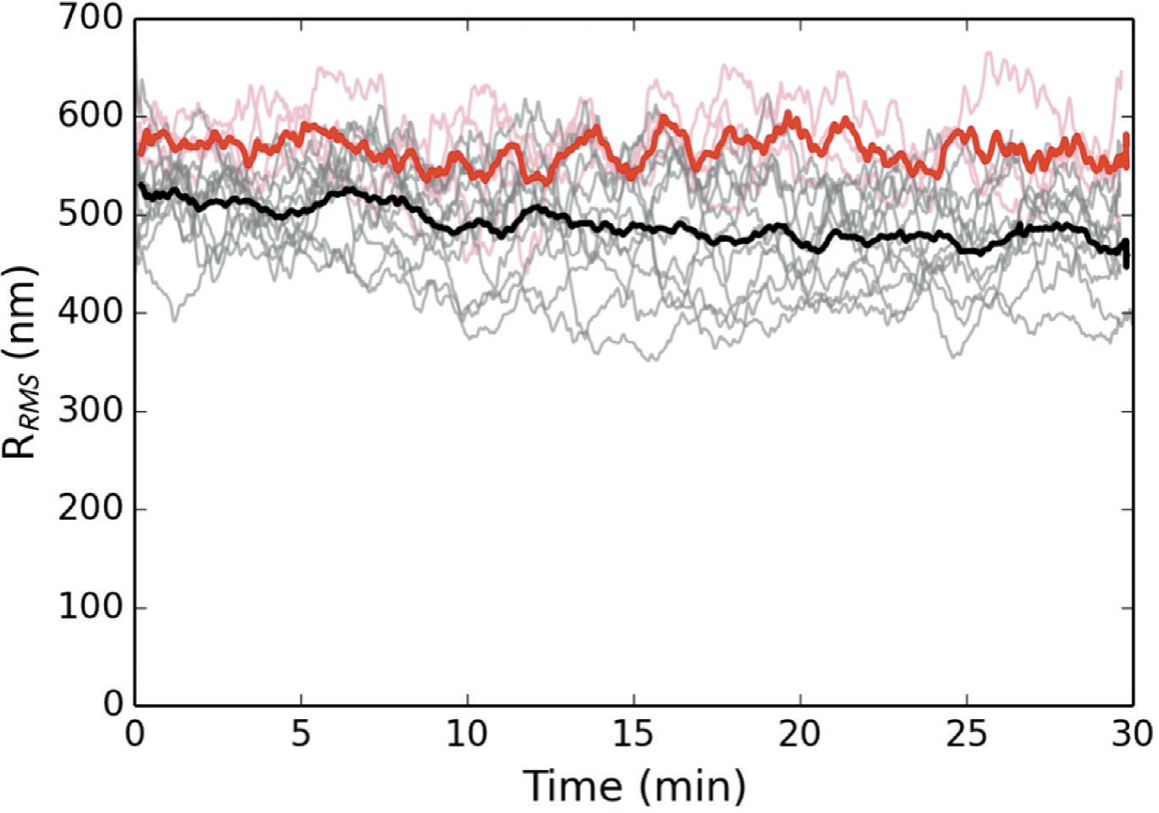
Dynamics of DNA bound by H-NS and the mutant Hha_(R14A/R17A)._ The black curves display the RMS excursion of the tethered microspheres as a function of time after release from the optical trap. The red curves are a control absent an applied force. Both proteins are at a concentration of 200 nM. The prominent solid curves are averaged over the respective trajectories for each population. All trajectories have been smoothed by a 60 s running window. While the mutant protein tends to reduce the RMS observed, the protein–DNA complex can no longer be triggered to collapse.

Hha contains several surface exposed, positively charged residues that we have shown are critical for function (Figure 6a). The Hha mutant R14A/R17A, which lacks several of these residues, folds properly, associates with H-NS normally, but fails to augment gene silencing *in vivo*. Given the position of the charged residues in the complex, and their critical role in gene silencing, we previously postulated that these residues contact DNA. In this work, we have assessed whether the Hha_(R14A/R17A)_ mutant maintained the ability to induce collapse of the H-NS–DNA complex and found that it does not. Figure 4 shows several trajectories for DNA bound by the H-NS/Hha_(R14A/R17A)_ complex, and is seen to remain stable for at least 30 min post application of a mechanical tension that caused the DNA bound by the H-NS/Hha complex to collapse. These results suggest that the positively charged surface of Hha plays a role in overcoming electrostatic repulsion of adjacent DNA segments thereby enabling H-NS to effectively collapse DNA.

**Figure 5.**
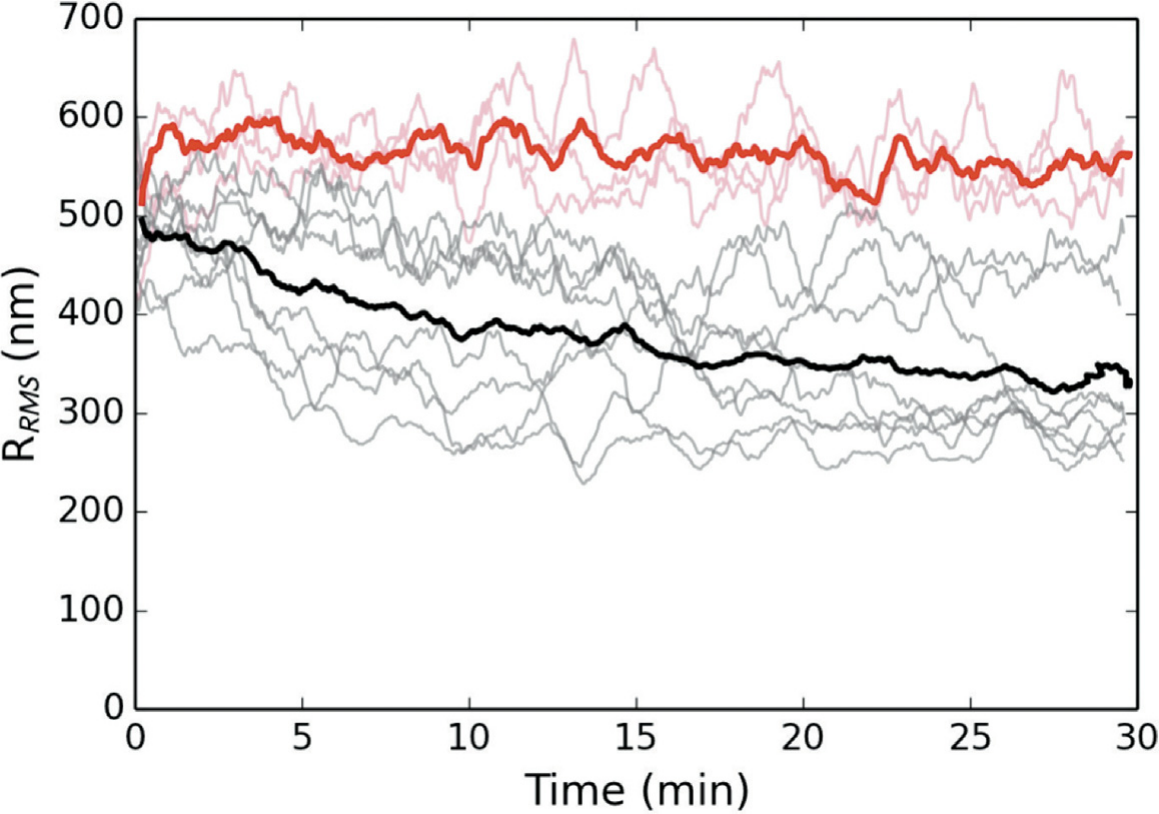
Dynamics of DNA bound by 200 nM of H-NS and 2 mM of MgCl_2_ as a function of time. The black curves display the RMS excursion of the tethered microspheres as a function of time after release from the optical trap. The red curves are a control absent an applied force. The prominent solid curves are averaged over the respective trajectories for each population. All trajectories have been smoothed by a 60 s running window. Notice that the DNA, bound by H-NS alone, can be triggered to collapse in the presence of millimolar concentrations of Mg^2+^.

**Figure 6.**
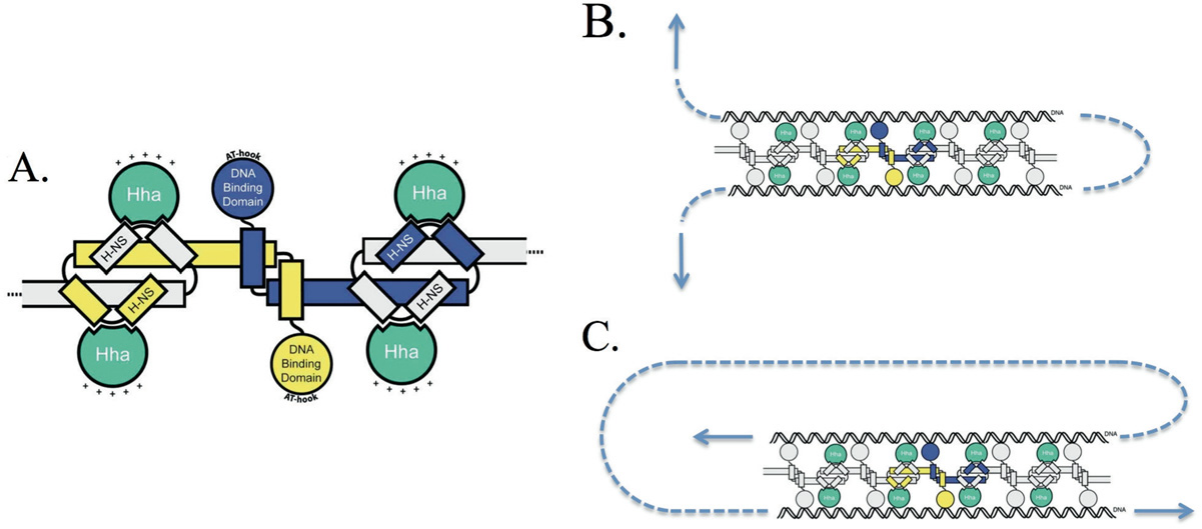
(**A**) Schematic of an H-NS oligomer in complex with Hha (the positively charged surface of Hha is indicated by ‘+’). (**B**) By dissociating one protein at a time, this bridging configuration should be relatively sensitive to an applied force (indicated by the arrows). (**C**) An alternative orientation that would be much more stable to dissociation by an applied tension.

Divalent salts such as Mg^2+^ can significantly soften DNA and could also potentially neutralize charges on the DNA backbone. We assessed whether the addition of Mg^2+^ could effectively substitute for Hha in facilitating the collapse of DNA by H-NS. Indeed, we find that we are able to trigger a collapse in DNA bound by H-NS at between 1 to 2 mM of Mg^2+^ (Figure 5), which is similar to the previously reported threshold where H-NS goes from stiffening to bridging DNA (22). We also observe that at still higher concentrations of Mg^2+^ (10–20 mM), the DNA bound by H-NS will spontaneously collapse even without an applied force.

## DISCUSSION

H-NS is known to form extended filaments along DNA, which can result in a significant increase in persistence length. When we apply force to the H-NS/DNA complex, we appear to soften the filaments; perhaps by altering how either H-NS is bound to the DNA or to other H-NS molecules. It is known that these filaments, which have a vacant series of DNA binding domains, are capable of forming bridges between strands of DNA. The protein–DNA complex is capable of bridging with itself, but initially the complex may be too stiff for distant segments to come in sufficient contact for self-bridging to occur. After force is applied, the complex becomes significantly more flexible, but we suspect that the negative charge of the DNA phosphate backbone causes an electrostatic repulsion that again prevents bridging. However, when the filaments are bound by Hha, the net positive charge of the cofactor acts to screen the electrostatic repulsion of the backbone, which allows the protein–DNA complex to bridge if pre-stressed, leading to the dynamical condensation of the DNA that we have witnessed.

When we replace Hha with the mutant Hha_(R14A/R17A)_, which is known to abolish transcriptional silencing effects of H-NS *in vivo*, the complex can no longer be induced to collapse. This suggests that our single-molecule observations correlate well with the results obtained *in vivo* and that the collapsed structure may represent the functional silencing complex. Hha_(R14A/R17A)_ is missing two exposed charged residues so these observations are in agreement with our charge screening hypothesis. We also expect that by adding divalent salts such as Mg^2+^ to the buffer, we would observe collapse phenomena in H-NS bound filaments unassisted by Hha, which is exactly what we witness (at 2 mM). In this case, the collapse is much more erratic and at even higher concentrations of Mg^2+^ (10–20 mM), the H-NS/DNA complex spontaneously collapses even without applying force, which is again consistent since multivalent ions like Mg^2+^ are known to also soften bare dsDNA (31) (in our experiment, bare dsDNA in the presence of these concentrations of Mg^2+^ simply results in a slightly shorter RMS extension). The range of Mg^2+^ concentrations where we see DNA collapse is nearly identical to where other groups have seen a transition between ‘bridging’ and ‘stiffening’ behaviors in H-NS nucleoprotein complexes (22). In contrast to the collapsed form generated by the H-NS/Hha complex, we are able to reverse the collapse of DNA bound by H-NS alone in the presence of Mg^2+^, which is also in agreement with previous results where Mg^2+^ induced bridging was disrupted by picoNewton forces (21). As a final note, we observed that increasing the monovalent salt KCl concentration from 50 to 150 mM had little effect on our results.

H-NS dimers at high concentrations may multimerize to form superhelical filaments in the absence of DNA (16). This twisted filamentary structure suggests that the condensed DNA state that H-NS adopts in the presence of Hha may be that of a tightly coiled solenoid (16). In fact, after collapse, we are unable to extend the condensed DNA with our optical tweezers suggesting that self-bridging is induced at multiple sites. We suspect that the proteins are bridging the DNA in an orientation similar to that displayed in Figure 6c. A force imposed along the DNA would have to dissociate multiple proteins to disrupt the loop. An orientation such as that shown in Figure 6b, however, would be much more susceptible to an applied force as the complex could be ‘unzipped’ one protein at a time. Note, however, we see no evidence that H-NS, when bound to DNA in the absence of Hha, imposes a rigid superhelical structure upon the DNA as the contour length of the protein–DNA complex does not seem to be altered from the bare DNA contour length.

Our results imply that the Hha/H-NS complex may selectively condense bacterial DNA based upon the level of mechanical tension experienced along different regions of the chromosome. A question remains as to whether the behavior we witness in our *in vitro* experiments could have physiological significance within a live bacterial cell; in particular, what could give rise to a mechanical impulse leading to condensation of the chromosomal DNA? Compelling evidence from both micro-rheological *in vitro* and *in vivo* experiments and analytical models suggest that the procession of molecular motors, like Myosin, along cytoskeletal filaments-a process that continually consumes ATP (or GTP)-generates additional fluctuations in excess of thermal fluctuations within Eukaryotes (32). In fact, similar phenomena have been observed in Prokaryotes. Driven in this case by enzymatic activity, active ATP dependent fluctuations were found to significantly contribute to the motion of chromosomal loci in live bacteria (33). Effects of cellular crowding and ATP driven fluctuations suggest that the intracellular environment should regularly interact with DNA, giving rise to mechanical fluctuations in tension along the chromosome. These fluctuations may provide biomechanical pathways for regulating gene expression and cellular function complementary to conventional biochemical pathways (34).

## SUPPLEMENTARY DATA

Supplementary Data are available at NAR Online.

SUPPLEMENTARY DATA

## References

[1] Ochman H., Lawrence J.G., Groisman E.A. (2000). Lateral gene transfer and the nature of bacterial innovation. Nature.

[2] Gevers D., Cohan F.M., Lawrence J.G., Spratt B.G., Coenye T., Feil E.J., Stackebrandt E., Van de Peer Y., Vandamme P., Thompson F.L. (2005). Opinion: re-evaluating prokaryotic species. Nat. Rev. Microbiol..

[3] Jain R., Rivera M.C., Moore J.E., Lake J.A. (2002). Horizontal gene transfer in microbial genome evolution. Theor. Popul. Biol..

[4] Jun S., Wright A. (2010). Entropy as the driver of chromosome segregatioin. Nat. Rev. Microbiol..

[5] Thanbichler M., Wang S.C., Shapiro L. (2005). The bacterial nucleoid: a highly organized and dynamic structure. J. Cell. Biochem..

[6] Dorman C.J. (2013). Genome architecture and global gene regulation in bacteria: making progress towards a unified model. Nat. Rev. Microbiol..

[7] Luijsterburg M.S., Noom M.C., Wuite G.J., Dame R.T. (2006). The architectural role of nucleoid-associated proteins in the organization of bacterial chromatin: a molecular perspective. J. Struct. Biol..

[8] Maurer S., Fritz J., Muskhelishvili G. (2009). A systematic in vitro study of nucleoprotein complexes formed by bacterial nucleoid-associated proteins revealing novel types of DNA organization. J. Mol. Biol..

[9] Dame R.T. (2005). The role of nucleoid-associated proteins in the organization and compaction of bacterial chromatin. Mol. Microbiol..

[10] Navarre W.W., Porwollik S., Wang Y., McClelland M., Rosen H., Libby S.J., Fang F.C. (2006). Selective silencing of foreign DNA with low GC content by the H-NS protein in Salmonella. Science.

[11] Navarre W.W., McClelland M., Libby S.J., Fang F.C. (2007). Silencing of xenogeneic DNA by H-NS-facilitation of lateral gene transfer in bacteria by a defense system that recognizes foreign DNA. Genes Dev..

[12] Lucchini S., Rowley G., Goldberg M.D., Hurd D., Harrison M., Hinton J.C. (2006). H-NS mediates the silencing of laterally acquired genes in bacteria. PLoS Pathog..

[13] Oshima T., Ishikawa S., Kurokawa K., Aiba H., Ogasawara N. (2006). Escherichia coli histone-like protein H-NS preferentially binds to horizontally acquired DNA in association with RNA polymerase. DNA Res..

[14] Grainger D.C., Hurd D., Goldberg M.D., Busby S.J. (2006). Association of nucleoid proteins with coding and non-coding segments of the Escherichia coli genome. Nucleic Acids Res..

[15] Ali S.S., Xia B., Liu J., Navarre W.W. (2012). Silencing of foreign DNA in bacteria. Curr. Opin. Microbiol..

[16] Arold S.T., Leonard P.G., Parkinson G.N., Ladbury J.E. (2010). H-NS forms a superhelical protein scaffold for DNA condensation. Proc. Natl. Acad. Sci. U.S.A..

[17] Ueguchi C., Suzuki T., Yoshida T., Tanaka K., Mizuno T. (1996). Systematic mutational analysis revealing the functional domain organization of Escherichia coli nucleoid protein H-NS. J. Mol. Biol..

[18] Shindo H., Iwaki T., Ieda R., Kurumizaka H., Ueguchi C., Mizuno T., Morikawa S., Nakamura H., Kuboniwa H. (1995). Solution structure of the DNA binding domain of a nucleoid-associated protein, H-NS, from Escherichia coli. FEBS Lett..

[19] Amit R., Oppenheim A.B., Stavans J. (2003). Increased bending rigidity of single DNA molecules by H-NS, a temperature and osmolarity sensor. Biophys. J..

[20] Dame R.T., Noom M.C., Wuite G.J. (2006). Bacterial chromatin organization by H-NS protein unravelled using dual DNA manipulation. Nature.

[21] Lim C.J., Lee S.Y., Kenney L.J., Yan J. (2012). Nucleoprotein filament formation is the structural basis for bacterial protein H-NS gene silencing. Sci. Rep..

[22] Liu Y., Chen H., Kenney L.J., Yan J. (2010). A divalent switch drives H-NS/DNA-binding conformations between stiffening and bridging modes. Genes Dev..

[23] De Alba C.F., Solorzano C., Paytubi S., Madrid C., Juarez A., Garcia J., Pons M. (2011). Essential residues in the H-NS binding site of Hha, a co-regulator of horizontally acquired genes in Enterobacteria. FEBS Lett..

[24] Garcia J., Madrid C., Cendra M., Juarez A., Pons M. (2009). N9L and L9N mutations toggle Hha binding and hemolysin regulation by Escherichia coli and Vibrio cholerae H-NS. FEBS Lett..

[25] Banos R.C., Vivero A., Aznar S., Garcia J., Pons M., Madrid C., Juarez A. (2009). Differential regulation of horizontally acquired and core genome genes by the bacterial modulator H-NS. PLoS Genet..

[26] Ali S.S., Whitney J.C., Stevenson J., Robinson H., Howell P.L., Navarre W.W. (2013). Structural insights into the regulation of foreign genes in Salmonella by the Hha/H-NS complex. J. Biol. Chem..

[27] Segall D., Nelson P., Phillips R. (2006). Volume-exclusion effects in tethered-particle experiments: bead size matters. Phys. Rev. Lett..

[28] Marko J.F., Siggia E.D. (1995). Stretching DNA. Macromolecules.

[29] Yin H., Landick R., Gelles J. (1994). Tethered particle motion method for studying transcript elongation by a single RNA polymerase molecule. Biophys. J..

[30] Finzi L., Gelles J. (1995). Measurement of lactose repressor-mediated loop formation and breakdown in single DNA molecules. Science.

[31] Baumann C.G., Smith S.B., Bloomfield V.A., Bustamante C. (1997). Ionic effects on the elasticity of single DNA molecules. Proc. Natl. Acad. Sci. U.S.A..

[32] Brangwynne C.P., Koenderink G.H., Mackintosh F.C., Weitz D.A. (2008). Nonequilibrium microtubule fluctuations in a model cytoskeleton. Phys. Rev. Lett..

[33] Weber S.C., Spakowitz A.J., Theriot J.A. (2012). Nonthermal ATP-dependent fluctuations contribute to the in vivo motion of chromosomal loci. Proc. Natl. Acad. Sci. U.S.A..

[34] Milstein J.N., Meiners J.C. (2011). On the role of DNA biomechanics in the regulation of gene expression. J. R. Soc. Interface.

