# Supplementary material for: A biomechanical mechanism for initiating DNA packaging
Source: Nucleic Acids Res. 2014 Oct 1;42(19):11921–7. doi: 10.1093/nar/gku896 (PMC4231757; doi:10.1093/nar/gku896)
Supplement: SUPPLEMENTARY DATA [file supp_42_19_11921__index.html]

A biomechanical mechanism for initiating DNA packaging — A biomechanical mechanism for initiating DNA packaging — SUPPLEMENTARY DATA 

# A biomechanical mechanism for initiating DNA packaging

## SUPPLEMENTARY DATA

**Files in this Data Supplement:**

- SUPPLEMENTARY DATA
