# Supplementary material for: A biomechanical mechanism for initiating DNA packaging
Source: Nucleic Acids Res. 2014 Oct 1;42(19):11921–7. doi: 10.1093/nar/gku896 (PMC4231757; doi:10.1093/nar/gku896)
Supplement: SUPPLEMENTARY DATA [file supp_gku896_nar-02344-f-2014-File008.pdf]

Supplementary Materials

A Biomechanical Mechanism for Initiating DNA Packaging

Haowei Wang[1], Sam Epstein[1,2], Sabrina S. Ali[3], William Wiley Navarre[3] and Joshua N. Milstein[1,2]

[1]Department of Chemical and Physical Sciences, University of Toronto Mississauga, Mississauga, ON L5L 1C6, CAN
[2]Department of Physics, University of Toronto, Toronto, ON M5S 1A7, CAN
[3]Department of Molecular Genetics, University of Toronto, Toronto, ON M5S 1A8, CAN

I. Heating Effect of the Trap Laser

One might worry that the laser is heating the protein and somehow altering its function. Since we still observe the collapse behavior at the low powers necessary to apply ~1 pN of force, this seems highly unlikely. Still, we did a simple test on the stability of H-NS, which was to briefly heat the protein on a hot plate before adding it to our chamber (up to 57°C, which is much higher than we could imagine the laser is able to locally heat the sample). The protein was still able to induce a collapse in the DNA.

II. Fitting the contour length (L_C) and persistence length (ξ) to the WLC

The adjusted R^2 provides a measure to how well the inclusion of an additional parameter improves the match of a model to data. Assuming n is the number of data points and m is the number of regression coefficients, then the adjusted R^2 is equal to

$$R_{adj}^2 = 1 - \frac{SSE \times (n-1)}{SST \times (n-m)}. \qquad (3)$$

In the above equation, $SSE = \sum_{i=1}^{n}(y_i - \hat{y}_i)^2$ is the sum of squared error and $SST = \sum_{i=1}^{n}(y_i - \bar{y})^2$ is the sum of squared total, y_i is the measured data, $\hat{y}_i$ is the fitted value at each point and $\bar{y}$ is the mean of the data. When comparing two models, the model with an adjusted R^2 value closer to 1 is the more appropriate one.

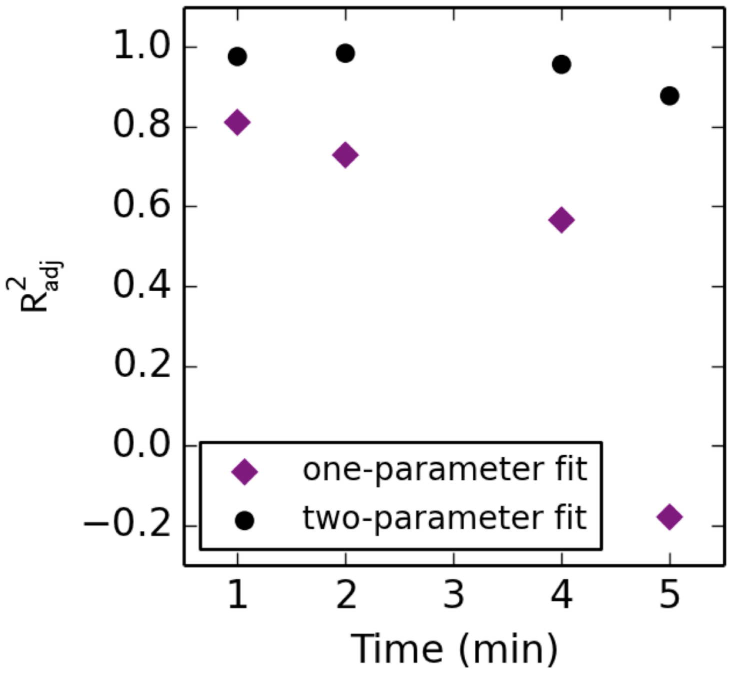

Figure S1. Adjusted R^2 values of WLC fitting with a single parameter ξ (diamond) and two parameters ξ and L_C (circle) during collapse of the DNA bound by H-NS/Hha.

Figure S1 compares a single parameter fit (ξ) to a two parameter fit (ξ and L_C) of our force-extension data during the collapse of DNA bound by H-NS/Hha (Fig. 1). A measurement taken one minute after centering the DNA within the optical trap shows a clear improvement to the fit if the contour length is added as an additional fit parameter. After 5 minutes, a single parameter WLC model no longer fits the data at all, but gives a reasonable fit if the maximum contour length is adjusted.

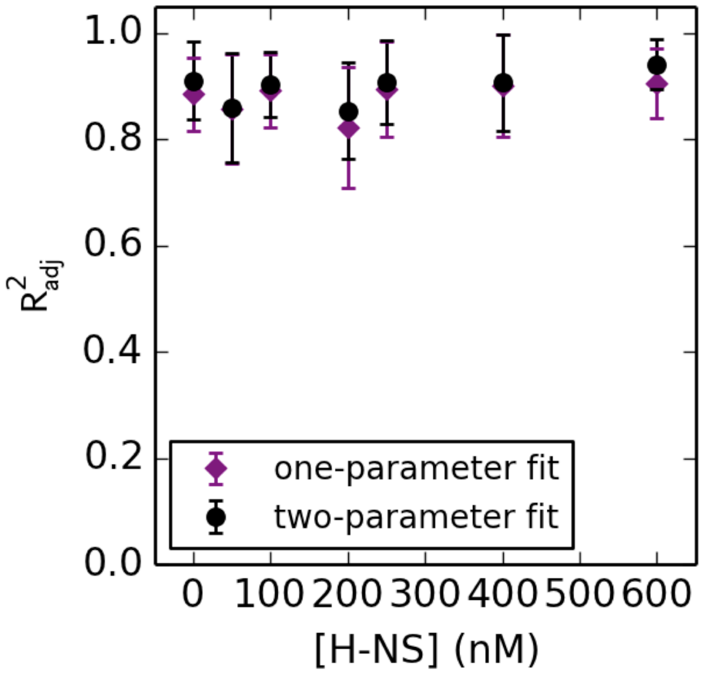

Figure S2. Adjusted R^2 values of WLC fitting with a single parameter ξ (diamond) and two parameters ξ and L_C (circle) for DNA bound by an increasing concentration of H-NS. The error bars show the standard deviation in the adjusted R^2 values over multiple force-extension measurements.

Figure S2 compares a single parameter fit (ξ) to a two parameter fit (ξ and L_C) of our force-extension data for DNA bound by an increasing concentration of H-NS (Fig. 3). From the error analysis, we find no motivation to include L_C as an additional fitting parameter. This is another indication that the contour length of the DNA, when bound solely by H-NS, remains unaffected.

III. Statistics of Softening and Collapse*

Figure 1:

[H-NS/Hha] (nM)	# Beads (Collapse)	# Beads (Control)
**100**	11	6
**200**	14	11

Figure 3:

	[H-NS] (nM)	**0**	**50**	**100**	**200**	**250**	**400**	**600**
Pre-stressed	*ξ (nm)*	57	67	103	109	106	106	111
TPM	*Std. Error (nm)*	1	1	8	1	2	2	4
	# of Beads	126	27	16	16	7	4	3
Tweezers	*ξ (nm)*	54	57	62	69	70	68	63
	Std. Error (nm)	1	3	5	2	4	4	4
	# of Beads	96	5	10	28	2	15	30
Post stressed	*ξ (nm)*	53	56	66	51	59	66	67
TPM	*Std. Error (nm)*	3	2	4	2	6	6	6
	# of Beads	3	5	2	2	3	6	3

Figures 4 and 5:

	# Beads (Collapse)	# Beads (Control)
**$\mathbf{Hha}_{(R14A/R17A)}$**	11	4
**$\mathbf{Mg^{2+}}$**	8	4

*Note: for the # Beads (Control) presented, we provide the number of traces shown in the respective figure. Since all working tethers within a field of view are traced during each collapse, we have a large amount of data of the molecules displaying stable behavior in the absence of a mechanical force.
